# A simulation study of regression approaches for estimating risk ratios in the presence of multiple confounders

**DOI:** 10.1186/s12982-021-00107-2

**Published:** 2021-12-11

**Authors:** Kanako Fuyama, Yasuhiro Hagiwara, Yutaka Matsuyama

**Affiliations:** 1 Graduate School of Interdisciplinary Information Studies, The University of Tokyo, Tokyo, Japan; 2 Department of Biostatistics, School of Public Health, The University of Tokyo, 7-3-1, Hongo, Bunkyo-ku, 113-0033 Tokyo, Japan

**Keywords:** Risk ratio, Logistic regression, Modified Poisson regression, Standardization, Confounding, Simulation study

## Abstract

**Background:**

Risk ratio is a popular effect measure in epidemiological research. Although previous research has suggested that logistic regression may provide biased odds ratio estimates when the number of events is small and there are multiple confounders, the performance of risk ratio estimation has yet to be examined in the presence of multiple confounders.

**Methods:**

We conducted a simulation study to evaluate the statistical performance of three regression approaches for estimating risk ratios: (1) risk ratio interpretation of logistic regression coefficients, (2) modified Poisson regression, and (3) regression standardization using logistic regression. We simulated 270 scenarios with systematically varied sample size, the number of binary confounders, exposure proportion, risk ratio, and outcome proportion. Performance evaluation was based on convergence proportion, bias, standard error estimation, and confidence interval coverage.

**Results:**

With a sample size of 2500 and an outcome proportion of 1%, both logistic regression and modified Poisson regression at times failed to converge, and the three approaches were comparably biased. As the outcome proportion or sample size increased, modified Poisson regression and regression standardization yielded unbiased risk ratio estimates with appropriate confidence intervals irrespective of the number of confounders. The risk ratio interpretation of logistic regression coefficients, by contrast, became substantially biased as the outcome proportion increased.

**Conclusions:**

Regression approaches for estimating risk ratios should be cautiously used when the number of events is small. With an adequate number of events, risk ratios are validly estimated by modified Poisson regression and regression standardization, irrespective of the number of confounders.

**Supplementary Information:**

The online version contains supplementary material available at 10.1186/s12982-021-00107-2.

## Background

A cohort study is a type of observational study that aids in evaluating associations between exposures and outcomes. In such studies, regression analysis is frequently used to estimate the effect of exposure adjusted for multiple confounders. For binary outcomes, logistic regression has been widely employed to estimate adjusted odds ratios. Because of interpretation difficulties, odds ratios are often interpreted as approximates of risk ratios under the assumption of rare events [1]. Although the odds ratio approximates the risk ratio if the outcome risk is sufficiently low for all study subjects, when some of the subjects have risk higher than 10%, the odds ratio is known to be distorted away from the null value compared to the risk ratio [2, 3].

Among the generalized linear models, log-binomial regression models can be used to directly estimate adjusted risk ratios for both common and rare events [4]. However, log-binomial regression using the standard maximum likelihood estimation method often fails to converge [5, 6]. To solve this problem, modified Poisson regression has been proposed [7] and has been applied to estimate adjusted risk ratios in numerous epidemiologic studies (e.g., [8–12]). Moreover, regression standardization using logistic regression could be another possible workaround [13, 14].

In terms of confounding adjustment using regression analysis, the number of confounders that can be included in regression models has been investigated in relation to the number of events or subjects [15–19]. Although a simple criterion of ten or more events per variable is well known for logistic regression [20, 21], other factors such as the number of events and confounders per se and the effect sizes are reported to influence the valid estimation of adjusted odds ratios [17–19]. On the other hand, the statistical performance of risk ratio estimation has not been well examined in the presence of multiple confounders.

This study sought to evaluate statistical performance in the presence of multiple confounders of the three regression approaches for estimating risk ratios: (1) risk ratio interpretation of logistic regression coefficients, (2) modified Poisson regression, and (3) regression standardization using logistic regression. After briefly summarizing approaches for estimating risk ratios, we evaluate the statistical performance of the three approaches by means of simulation. We then discuss the interpretation of the simulation results and draw conclusions about regression approaches for estimating risk ratios in the presence of multiple confounders.

## Methods

### Regression approaches for estimating risk ratios

We consider a cohort study of *n* subjects involving binary outcome *Y*_*i*_ (1 for event and 0 for no event), binary exposure *A*_*i*_ (1 for exposure and 0 for no exposure), and column vector of confounders *L*_*i*_ for each subject *i*. Logistic regression is commonly used to control for confounders and assess the influence of exposure for this type of data. The logistic regression model with first-order terms of exposure and confounders is expressed as follows (we assume that the regression models are correctly specified below unless otherwise noted):

$$\text{log}\frac{E\left({Y}_{i}|{A}_{i},{L}_{i};\alpha \right)}{1-E\left({Y}_{i}|{A}_{i},{L}_{i};\alpha \right)}={\alpha }_{0}+{\alpha }_{1}{A}_{i}+{\alpha }_{2}^{T}{L}_{i}={X}_{i}^{T}\alpha ,$$

where $\alpha ={\left({\alpha }_{0},{\alpha }_{1},{\alpha }_{2}^{T}\right)}^{T}$ is the unknown parameter vector, and ${X}_{i}={\left(1,{A}_{i},{L}_{i}^{T}\right)}^{T}$. Under this model, the exponentiated exposure coefficient, $\text{e}\text{x}\text{p}\left({\alpha }_{1}\right)$, indicates the adjusted odds ratio, which is often interpreted as the risk ratio under the assumption of rare events [1]. Assuming that *Y*_*i*_ follows a binomial distribution given *A*_*i*_ and *L*_*i*_, the parameter α is estimated using the standard maximum likelihood estimation method.

To estimate the risk ratio directly, the log-binomial regression model, the linear model of the log-transformed mean, may be straightforward [4]:

$$\text{log}E\left({Y}_{i}|{A}_{i},{L}_{i};\beta \right)={\beta }_{0}+{\beta }_{1}{A}_{i}+{\beta }_{2}^{T}{L}_{i}={X}_{i}^{T}\beta ,$$

where $\beta ={\left({\beta }_{0},{\beta }_{1},{\beta }_{2}^{T}\right)}^{T}$ is the unknown parameter vector. Under this model, the exponentiated exposure coefficient, $\text{e}\text{x}\text{p}\left({\beta }_{1}\right)$, can be interpreted as the adjusted risk ratio without the assumption of rare events. Assuming that *Y*_*i*_ follows a binomial distribution given *A*_*i*_ and *L*_*i*_, the standard maximum likelihood estimation method yields a consistent and asymptotically efficient estimator for *β*. However, in real-world applications, the iterative procedures for log-binomial regression models often fail to converge [5, 6] and were thus excluded from our simulation experiments.

One proposed solution, modified Poisson regression, estimates parameter *β* of the log-binomial regression model by solving the following estimating equations for *β* [7]:

$$U\left(\beta \right)=\sum _{i=1}^{n}{X}_{i}\left\{{Y}_{i}-\text{exp}\left({X}_{i}^{T}\beta \right)\right\}=0.$$

The estimating equations of the modified Poisson regression are equivalent to the score equations of the Poisson regression. The estimator for the standard error is obtained from the robust sandwich variance estimator:

$$\begin{aligned} \widehat{Var}\left(\widehat{\beta }\right) ={\left\{\sum
_{i=1}^{n}{X}_{i}\text{exp}\left({X}_{i}^{T}\widehat{\beta }\right){{X}_{i}}^{T}\right\}}^{-1}\left[\sum
_{i=1}^{n}{X}_{i}{\left\{{Y}_{i}-\text{exp}\left({X}_{i}^{T}\widehat{\beta }\right)\right\}}^{2}{{X}_{i}}^{T}\right]{\left\{\sum_{i=1}^{n}{X}_{i}\text{exp}\left({X}_{i}^{T}\widehat{\beta }\right){{X}_{i}}^{T}\right\}}^{-1}. \end{aligned}$$

The estimator obtained from the estimating equation is consistent and asymptotically normal, albeit without asymptotic efficiency. For rare events, the modified Poisson regression estimators approximate maximum likelihood estimators of log-binomial and logistic regression, and the efficiency loss should be small [22]. Previous simulation results suggest that the modified Poisson regression estimates are generally close to the maximum likelihood counterparts [7, 23]. The modified Poisson regression is also reported to be less sensitive to outliers [24] and less biased when the mean structure is misspecified [25]. Potential predicted probabilities above 1 [26, 27] are not fatal if the analysis aims to estimate the adjusted risk ratio and not the individual predicted probabilities.

Another approach for estimating the risk ratio is regression standardization using logistic regression [13, 14]. Instead of directly interpreting the logistic regression coefficients, the risk ratio among the entire population is calculated based on predicted probabilities estimated from logistic regression, which are constrained to fall between 0 and 1. Using maximum likelihood estimates of logistic regression $\widehat{\alpha }$, the predicted risk if subject *i* was exposed is given by

$${\widehat{\mu }}_{i1}=\frac{\text{e}\text{x}\text{p}\left({X}_{i1}^{T}\widehat{\alpha }\right)}{1+\text{e}\text{x}\text{p}\left({X}_{i1}^{T}\widehat{\alpha }\right)},$$

where ${X}_{i1}={\left(1, 1,{L}_{i}^{T}\right)}^{T}$, and the predicted risk if subject *i* was not exposed is given by

$${\widehat{\mu }}_{i0}=\frac{\text{e}\text{x}\text{p}\left({X}_{i0}^{T}\widehat{\alpha }\right)}{1+\text{e}\text{x}\text{p}\left({X}_{i0}^{T}\widehat{\alpha }\right)},$$

where ${X}_{i0}={\left(1, 0,{L}_{i}^{T}\right)}^{T}$. The risk ratio for exposure is computed by taking the ratio of these risks averaged over the population:

$$\widehat{\text{R}\text{R}}=\frac{\sum _{i=1}^{n}{\widehat{\mu }}_{i1}}{\sum _{i=1}^{n}{\widehat{\mu }}_{i0}}.$$

The estimator for the standard error of $\text{log}\widehat{\text{R}\text{R}}$ is easily obtained using the delta method [28]:

$$\widehat{Var}\left(\text{log}\widehat{\text{R}\text{R}}\right)={R}^{T}\widehat{Var}\left(\widehat{\alpha }\right)R,$$

where

$$\begin{aligned}R= & \frac{1}{\sum _{i=1}^{n}{\widehat{\mu }}_{i1}}\left[\sum
_{i=1}^{n}\frac{\text{e}\text{x}\text{p}\left({X}_{i1}^{T}\widehat{\alpha
}\right)}{{\left\{1+\text{e}\text{x}\text{p}\left({X}_{i1}^{T}\widehat{\alpha }\right)\right\}}^{2}}{X}_{i1}\right]\\ & - \frac{1}{\sum _{i=1}^{n}{\widehat{\mu }}_{i0}}\left[\sum
_{i=1}^{n}\frac{\text{e}\text{x}\text{p}\left({X}_{i0}^{T}\widehat{\alpha
}\right)}{{\left\{1+\text{e}\text{x}\text{p}\left({X}_{i0}^{T}\widehat{\alpha
}\right)\right\}}^{2}}{X}_{i0}\right],\end{aligned}$$

and $\widehat{Var}\left(\widehat{\alpha }\right)$ is the estimated variance-covariance matrix of logistic regression.

### Simulation methods

We conducted a simulation study to evaluate statistical performance in the presence of multiple confounders of three approaches for estimating risk ratios: (1) risk ratio interpretation of logistic regression coefficients, (2) modified Poisson regression, and (3) regression standardization using logistic regression. We simulated 270 scenarios (10,000 iterations) with settings varying systematically on sample size (2500, 5000, and 10,000), number of binary confounders (5, 10, and 20), exposure proportion (20% and 50%), risk ratio for exposure (1, 1.3, and 2), and outcome proportion (1%, 2%, 4%, 8%, and 16%). The simulation was carried out using SAS version 9.4 (SAS Institute, Inc.).

#### Data generation

We generated binary confounders, exposure, and outcome for each of the 2500, 5000, or 10,000 subjects. To create binary confounders, 5-, 10-, or 20-dimensional Gaussian variables with mean 0, variance 1, and pairwise correlations 0.33 were discretized into 0 and 1 at predefined points (Additional file 1: Table S1). The exposure was generated from a logistic regression model with first-order terms of confounders so that the specified exposure proportion (20% or 50%) was achieved on average (Additional file 1: Table S2). The outcome was generated from a log-binomial regression model with first-order terms of exposure and confounders using three different risk ratios for exposure (1, 1.3, or 2). Confounder-outcome associations were weakened for increased confounders so that the maximum possible individual risk did not exceed 1 at any number of confounders (Additional file 1: Table S3). We also conducted additional simulation experiments keeping the same moderate confounder-outcome associations regardless of the number of confounders. The parameter settings and the results of the additional simulation are provided in Additional file 2. The intercept was adjusted so that the specified outcome proportion (1%, 2%, 4%, 8%, or 16%) was achieved on average.

Although in some previous studies of logistic regression, the number of events was fixed across data sets assuming retrospective samplings such as in a case-control study [17, 19], we generated outcomes so that the specified proportion would be achieved only on average assuming prospective samplings such as in a cohort study. The expected number of events and events per confounder can be calculated using the sample size, outcome proportion, and the number of confounders (Table 1).

**Table 1:** The expected number of events and the expected number of events per confounder

Sample size	Outcome proportion	5 confounders		10 confounders		20 confounders	
		Events	EPC	Events	EPC	Events	EPC
2500	1%	25	5	25	2.5	25	1.25
	2%	50	10	50	5	50	2.5
	4%	100	20	100	10	100	5
	8%	200	40	200	20	200	10
	16%	400	80	400	40	400	20
5000	1%	50	10	50	5	50	2.5
	2%	100	20	100	10	100	5
	4%	200	40	200	20	200	10
	8%	400	80	400	40	400	20
	16%	800	160	800	80	800	40
10,000	1%	100	20	100	10	100	5
	2%	200	40	200	20	200	10
	4%	400	80	400	40	400	20
	8%	800	160	800	80	800	40
	16%	1600	320	1600	160	1600	80

*EPC* events per confounder

#### Analytical approaches

For each data set, we obtained the point estimate, standard error, and 95% Wald confidence interval of the log risk ratio for exposure from the three approaches. We performed logistic and modified Poisson regression with first-order terms of exposure and all confounders using the SAS GENMOD procedure. Note that the logistic regression misspecified the mean structure, and the degree of misspecification was larger with higher outcome proportions as more subjects had relatively high risks (Additional file 1: Fig. S1). We left the default settings unchanged for the optimization algorithm, starting values, and convergence criteria. If the algorithm for logistic regression was deemed to have converged based on criteria stated below, the results for the regression standardization were computed using the SAS program written by the authors.

#### Performance measures

For each scenario, we summarized the results in terms of convergence proportion, bias, Monte Carlo standard error, mean estimated standard error, and 95% confidence interval coverage for the log risk ratio for exposure. Because the software may falsely report convergence, giving invalid parameter estimates [29], the convergence of logistic and modified Poisson regression was evaluated based on the estimated standard errors of coefficients instead of the procedure’s reports. If the estimated standard error of any coefficient was missing, 0, or above 1000, the algorithm was deemed not to have converged. Results from the converged data sets were used to compute the following performance measures of the three approaches. To provide an intuitive understanding of bias, the mean estimated log risk ratio was transformed back to a linear scale. The Monte Carlo standard error was calculated as the standard deviation of the estimated log risk ratios. The mean estimated standard error was calculated as the average of the standard error estimates of the log risk ratio. The 95% confidence interval coverage was calculated as the proportion of estimated confidence intervals covering the true value.

## Results

### Convergence proportion

Nonconvergence mainly occurred when the sample size was 2500 and the outcome proportion was 1% (Additional file 1: Fig. S2). Under such scenarios, nonconvergence occurred for up to 2.3% of the data sets without identifiable trends associated with other factors such as the number of confounders, effect size, or exposure proportion. The convergence proportion and converged data sets were identical for logistic and modified Poisson regression. All data sets converged when the sample size was 2500 and the outcome proportion was higher than 2%, when the sample size was 5000 and the outcome proportion was higher than 1%, and when the sample size was 10,000. In the additional experiments, nonconvergence was more frequent with increased confounders (Additional file 2: Fig. S4).

### Bias

**Fig. 1:**
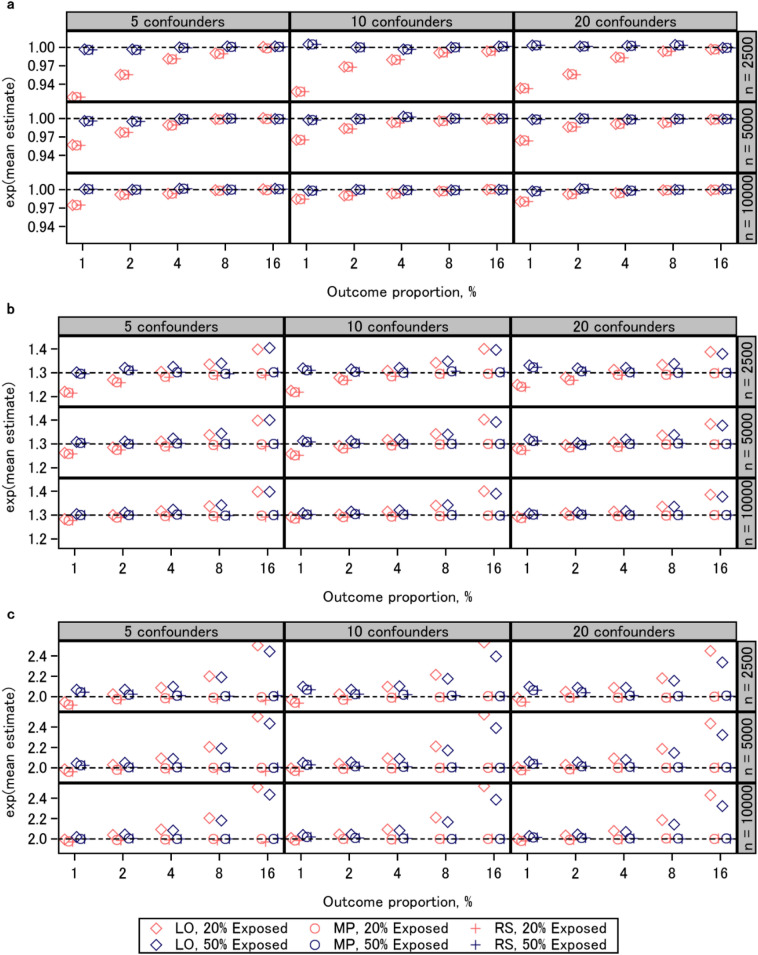
Mean estimated log risk ratio transformed back to linear scale. **a** risk ratio 1; **b** risk ratio 1.3; **c** risk ratio 2. *LO* logistic regression, *MP* modified Poisson regression, *RS* regression standardization

Figure 1 shows the results of biases for each scenario. In scenarios wherein true risk ratio was 1 (top), the direction and magnitude of the biases were comparable among the three approaches. When the outcome proportion was 1%, the three approaches underestimated the risk ratio with an exposure proportion of 20%. As the outcome proportion increased, the biases of the three approaches decreased. In scenarios wherein true risk ratio was 1.3 (middle) or 2 (bottom), the biases of the logistic regression coefficients showed a different trend compared to the others. With an exposure proportion of 20%, the three approaches underestimated the risk ratio when the outcome proportion was 1%. The largest bias amounted to approximately 25% of the true log risk ratio when the sample size was 2500. As the outcome proportion increased, the biases of the modified Poisson regression and regression standardization decreased, whereas overestimation biases were observed for logistic regression coefficients. With an exposure proportion of 50%, the three approaches overestimated the risk ratio when the outcome proportion was 1%. As the outcome proportion increased, the biases of the modified Poisson regression and regression standardization decreased, whereas the overestimation biases of logistic regression coefficients further increased. The number of confounders did not markedly affect the magnitude of bias. Although the biases associated with low outcome proportions were mitigated by the increased sample size, the biases of logistic regression coefficients associated with high outcome proportions were not. The results of biases were similar in the additional experiments (Additional file 2: Fig. S5).

### Standard error

**Fig. 2:**
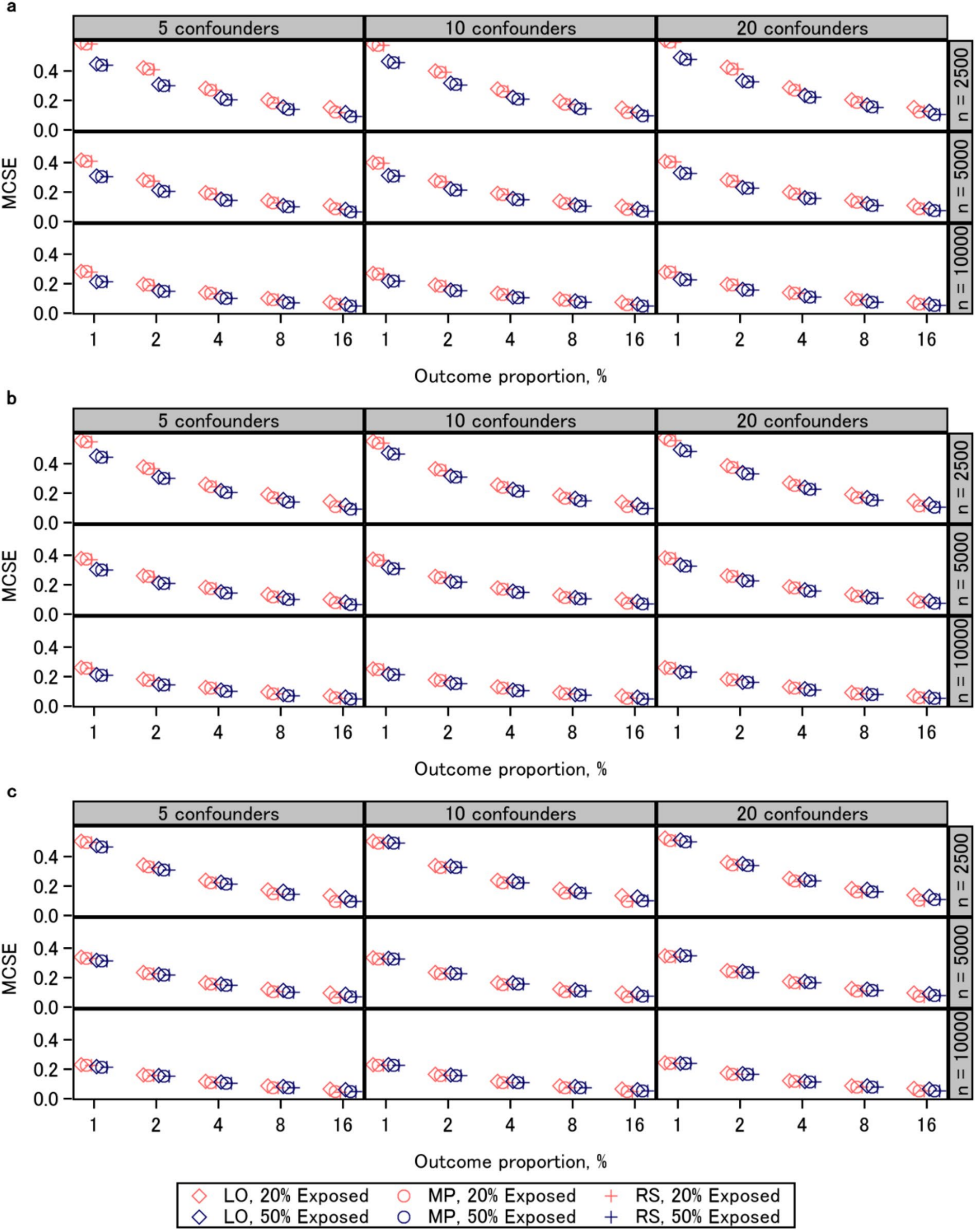
Monte Carlo standard error (MCSE). **a** risk ratio 1; **b** risk ratio 1.3; **c** risk ratio 2. *LO* logistic regression, *MP* modified Poisson regression, *RS* regression standardization

**Fig. 3:**
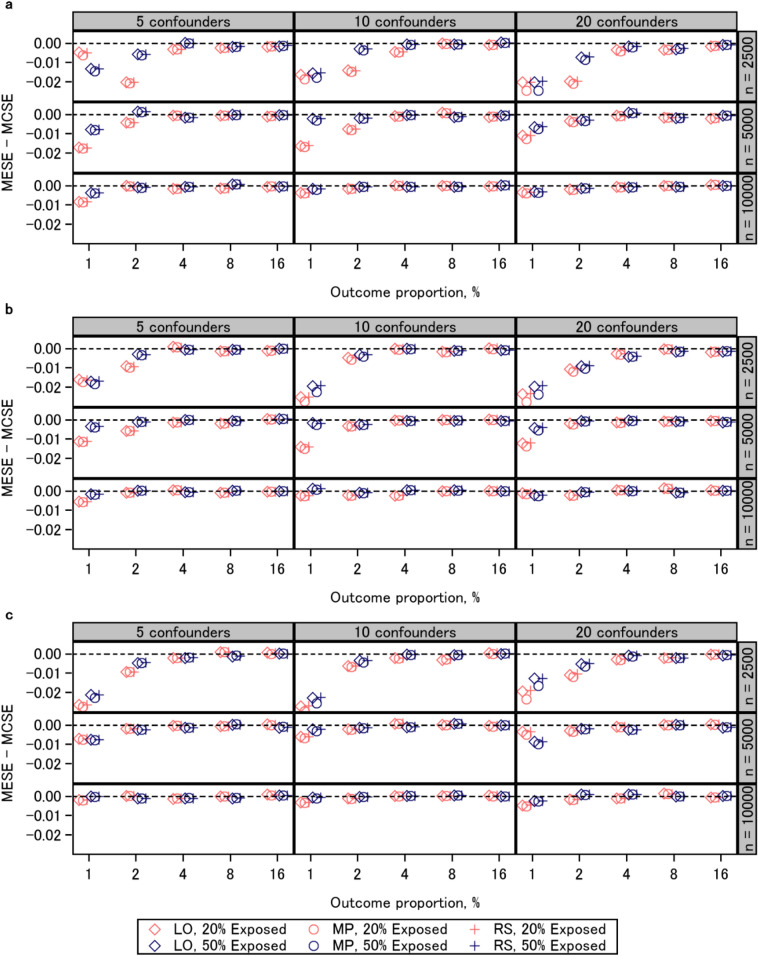
Mean estimated standard error (MESE) minus Monte Carlo standard error (MCSE). **a** risk ratio 1; **b** risk ratio 1.3; **c** risk ratio 2. *LO* logistic regression, *MP* modified Poisson regression, *RS* regression standardization

Figure 2 shows the results of the Monte Carlo standard error for each scenario. When the sample size was 2500 and the outcome proportion was lower than 4%, and when the sample size was 5000 and the outcome proportion was lower than 2%, the mean estimated standard error was slightly smaller than the Monte Carlo standard error for the three approaches, indicating that the three approaches underestimated the standard error (Fig. 3, Additional file 1: Fig. S3). When the outcome proportion was 2% or lower, the Monte Carlo standard error and mean estimated standard error were comparable among the three approaches. In contrast, when the outcome proportion was higher than 2%, those from the logistic regression coefficients were slightly larger than those from the other two approaches. The results of the Monte Carlo standard error, mean estimated standard error, and disparity thereof were associated with the expected number of events (Table 1). The results on standard error were similar in the additional experiments (Additional file 2: Figs. S6–S8).

### Coverage proportion

**Fig. 4:**
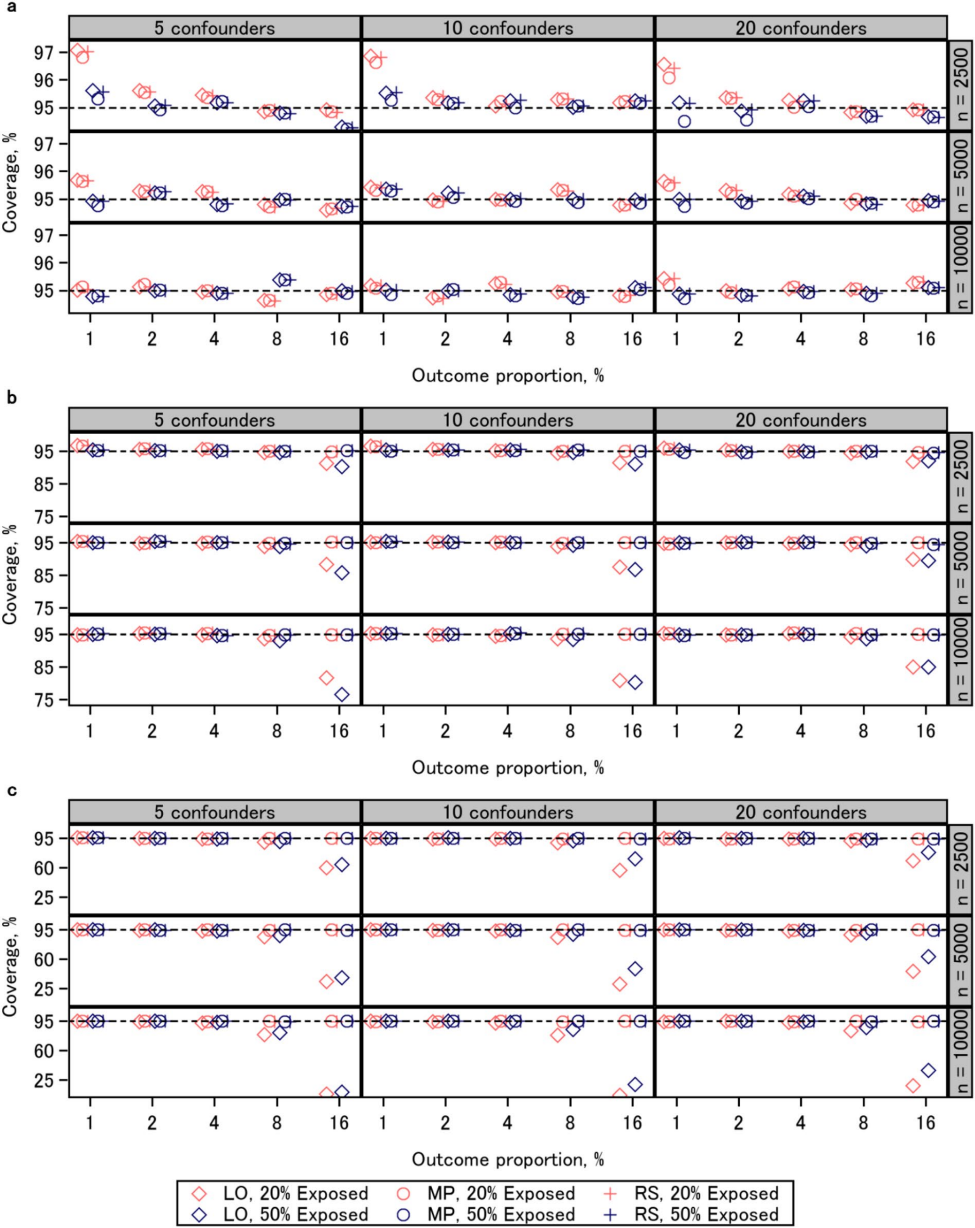
Coverage probability of the 95% Wald confidence interval. **a** risk ratio 1; **b** risk ratio 1.3; **c** risk ratio 2. *LO* logistic regression, *MP* modified Poisson regression, *RS* regression standardization

Figure 4 shows the results of 95% Wald confidence interval coverage for each scenario. In scenarios wherein true risk ratio was 1 (top), the coverage proportion was comparable among the three approaches. When the outcome proportion was 1%, overcoverage occurred for the three approaches, notably with a sample size of 2500 and an exposure proportion of 20%. As the outcome proportion increased, the coverage proportion became closer to the nominal level. In scenarios wherein true risk ratio was 1.3 (middle) or 2 (bottom), the coverage proportion of logistic regression coefficients showed a different trend compared to the others. When the outcome proportion was 1%, overcoverage occurred for the three approaches, notably with a sample size of 2500, an exposure proportion of 20%, and a risk ratio of 1.3. As the outcome proportion increased, the coverage proportion of the modified Poisson regression and regression standardization became closer to the nominal level, whereas undercoverage occurred for logistic regression coefficients. The number of confounders did not markedly affect the performance of confidence intervals. With other factors fixed, undercoverage of logistic regression associated with high outcome proportions was more severe with larger samples. The results of confidence interval coverage were similar in the additional experiments (Additional file 2: Fig. S9).

## Discussion

In this study, we evaluated the statistical performance of the regression approaches for estimating risk ratios in the presence of multiple confounders. In summary, with a sample size of 2500 and an outcome proportion of 1%, the three approaches were equally biased and yielded inaccurate confidence intervals. As the outcome proportion or sample size increased, modified Poisson regression and regression standardization yielded unbiased estimates with appropriate confidence intervals irrespective of the number of confounders. The risk ratio interpretation of logistic regression coefficients was substantially biased when the outcome proportion was relatively high and the true risk ratio was not 1. These results of the main simulation remained consistent in the additional simulation with different parameter settings for the outcome generation models.

In our simulation, nonconvergence occurred in identical data sets for logistic regression and modified Poisson regression. Logistic and Poisson regression may fail to converge due to separation or multicollinearity [30, 31]. Because we included multiple binary confounders in the models, quasi-complete separation was considered the dominant cause of nonconvergence. Although modified Poisson regression has been appreciated for being less prone to convergence issues compared to the log-binomial regression [7], it failed to converge for data sets wherein logistic regression failed to converge. In real-world applications, neither logistic nor modified Poisson regression is exempt from convergence issues.

When the outcome proportion was 1%, the three approaches were comparably biased (both upward and downward). It is well known that logistic regression may yield biased odds ratio estimates when the number of events is small and there are multiple confounders [15–19]. In such situations, one should also be careful with using modified Poisson regression and regression standardization. In our simulation, the magnitude of bias of these two approaches was associated with the expected number of events rather than the expected number of events per confounder; the increase in confounders for a fixed expected number of events did not markedly affect the magnitude of bias.

As the expected number of events increased corresponding to the increase in sample size or outcome proportion, modified Poisson regression and regression standardization yielded unbiased risk ratio estimates regardless of the number of confounders. In contrast, as the outcome proportion increased to 4% or higher, logistic regression coefficients were substantially upward biased except with true risk ratio 1. This is probably because the odds ratio no longer approximated the risk ratio. In our simulation, with a true risk ratio of 2 and an outcome proportion of 16%, the mean estimated logistic regression coefficients corresponded to an odds ratio of approximately 2.5, which may lead to an exaggeration of the exposure effect if interpreted as the risk ratio.

The three approaches underestimated the standard error when fewer than 100 events were expected (i.e., when the sample size was 2500 and the outcome proportion was lower than 4%, and when the sample size was 5000 and the outcome proportion was lower than 2%). Some previous simulation studies indicate that variance or standard error estimates of logistic regression may be unreliable when the number of events is small overall or relative to the number of confounders [15, 16]. Our simulation results suggest that similar problems in standard error estimation may arise from the different methods employed in our simulation. Several candidate methods are available for enhancing the performance of standard errors and the resulting confidence intervals. In modified Poisson regression, sandwich standard errors with small sample correction may be explored, as has been considered for linear regression [32] and modified least-squares regression for risk difference estimation [33, 34]. In regression standardization, the bootstrap method may be preferred for small sample sizes if the computational time is not critical [13, 35].

The Monte Carlo standard error and mean estimated standard error of the modified Poisson regression did not exceed those of the logistic regression. They were slightly larger for logistic regression with high outcome proportions, probably because of the larger point estimates. In theory, unlike maximum likelihood estimators, modified Poisson regression estimators are not asymptotically efficient. Nonetheless, our results suggest that efficiency loss in modified Poisson regression may be negligible for cohort studies involving rare outcomes; in such situations, the risk ratio interpretation of logistic regression coefficients will also hold good, however.

The three approaches yielded conservative 95% Wald confidence intervals when the expected number of events was 25 (i.e., when the outcome proportion was 1% and the sample size was 2500). Since this overcoverage was apparent concurrently with biased point estimates and underestimated standard errors, the overcoverage may have been caused by the non-normality of the estimated log risk ratios. These phenomena are in good agreement with a previous simulation study of logistic regression involving multiple binary confounders where coverage proportions were approximately 97% when 30 or fewer events were generated, often concurrently with biased point estimates [17]. Some caution is needed in interpreting the confidence intervals of the three approaches when the number of events is small; however, statistically significant results could be reliable because of conservatism.

As the expected number of events increased corresponding to the increase in sample size or outcome proportion, modified Poisson regression and regression standardization yielded appropriate confidence intervals regardless of the number of confounders. In contrast, as the outcome proportion increased to 8% or higher, undercoverage occurred for logistic regression coefficients except with true risk ratio 1, most likely because of the discrepancy between odds ratios and risk ratios. This undercoverage was more extreme with larger sample sizes, probably because the large sample size decreased the variability of the point estimates and the length of confidence intervals.

In our simulation, the three approaches were comparably biased and yielded inaccurate confidence intervals when only 25 events were expected. In such scenarios, the expected number of events per confounder varied between 1.25, 2.5, and 5. As long as a sufficient number of events were expected, modified Poisson regression and regression standardization yielded unbiased risk ratio estimates with appropriate confidence intervals regardless of the number of confounders. Specifically, when 50 or more events were expected, modified Poisson regression and regression standardization did not provide a problematic bias of over 15% of the true log risk ratio [17] in any of the scenarios with a risk ratio of 1.3 or 2, and the coverage of the two approaches fell within the range of 94–96% in all scenarios. This was the case even when the expected number of events per confounder was 2.5.

For logistic regression, the existing criteria and some previous simulation results emphasize the importance of the number of events per variable or confounder [15–19]. In contrast, according to our simulation, the performance of the modified Poisson regression and regression standardization were associated with the expected number of events per se. This result is in line with some previous simulation studies on logistic regression [17, 19]. Because simulation results for rare-outcome situations are greatly affected by convergence issues [36], further simulations including continuous confounders may help explore such criteria.

The risk ratio interpretation of logistic regression coefficients may be acceptable, assuming an adequate number of events, when the outcome proportion is low or the exposure effect is close to null. Nevertheless, the other two approaches performed equally well in such situations. Our results showed no relative merits in interpreting logistic regression coefficients as risk ratios. Of the other two approaches, modified Poisson regression may be simple and easy to implement, although the choice should ideally be based on the true mean structure expected from prior knowledge [37].

For odds ratio estimation using logistic regression, some authors have encouraged the use of propensity score analyses [16] and shrinkage techniques [19] in rare-outcome situations. These methods may also be helpful for the estimation of risk ratios when the number of events is small. Regression adjustment for the propensity score can be applied to modified Poisson regression. Shrinkage techniques may mitigate the sparse data bias of the predicted probabilities in regression standardization using logistic regression.

Our simulation study has some limitations. First, we generated outcomes from log-binomial regression models, assuming common risk ratios for all subjects. This means not only that the modified Poisson regression naturally outperforms the direct interpretation of logistic regression coefficients but also that our simulation procedures may have provided an edge to modified Poisson regression over regression standardization. Further research may help compare these approaches under different settings. Second, the distribution of individual risks and the overall outcome proportion will impact the discrepancy between odds ratios and risk ratios. Different distributions of individual risks may produce different results.

## Conclusions

In this study, we evaluated the statistical performance of the three regression approaches for estimating risk ratios in the presence of multiple confounders. Regression approaches for estimating risk ratios should be cautiously used when the number of events is small. With an adequate number of events, risk ratios are validly estimated by modified Poisson regression and regression standardization, irrespective of the number of confounders.

## Supplementary Information

**Additional file 1. **Simulation Experiments Presented in the Main Text.

**Additional file 2.** Additional Simulation Experiments.

## Data Availability

The codes used to generate the datasets are available from the corresponding author on reasonable request.
