# Supplementary material for: A simulation study of regression approaches for estimating risk ratios in the presence of multiple confounders
Source: Emerg Themes Epidemiol. 2021 Dec 11;18:18. doi: 10.1186/s12982-021-00107-2 (PMC8665581; doi:10.1186/s12982-021-00107-2)
Supplement: Supplementary file 1 — Additional file 1. Simulation Experiments Presented in the Main Text. [file 12982_2021_107_MOESM1_ESM.pdf]

Additional file 1 to "A Simulation Study of Regression Approaches for Estimating Risk Ratios in the Presence of Multiple Confounders"

Detailed Description of the Simulation Experiments Presented in the Main Text

Table S1. Parameter values for confounders in the simulation.

Number of Confounders	q																			
	L_1	L_2	L_3	L_4	L_5	L_6	L_7	L_8	L_9	L_{10}	L_{11}	L_{12}	L_{13}	L_{14}	L_{15}	L_{16}	L_{17}	L_{18}	L_{19}	L_{20}
5	0.4	0.5	0.7	0.8	0.4															
10	0.4	0.5	0.7	0.8	0.4	0.5	0.7	0.8	0.4	0.5										
20	0.4	0.5	0.7	0.8	0.4	0.5	0.7	0.8	0.4	0.5	0.7	0.8	0.4	0.5	0.7	0.8	0.4	0.5	0.7	0.8

Binary confounders were derived from 5-, 10-, or 20-dimensional Gaussian variables with mean 0, variance 1, and pairwise correlations 0.33. Each element up to Q(q) and above Q(q) was discretized into 0 and 1, respectively, where Q(.) is the quantile function of the normal distribution.

Table S2. Parameter values for exposure models in the simulation.

Number of Confounders	Odds ratio $\exp(\gamma_1)$ …, $\exp(\gamma_K)$																			
	L_1	L_2	L_3	L_4	L_5	L_6	L_7	L_8	L_9	L_{10}	L_{11}	L_{12}	L_{13}	L_{14}	L_{15}	L_{16}	L_{17}	L_{18}	L_{19}	L_{20}
5	2.5	0.5	1.5	0.67	1.1															
10	2.5	0.5	1.5	0.67	1.1	2.5	0.5	1.5	0.67	1.1										
20	2.5	0.5	1.5	0.67	1.1	2.5	0.5	1.5	0.67	1.1	2.5	0.5	1.5	0.67	1.1	2.5	0.5	1.5	0.67	1.1

The exposure models are logistic regression models: $\Pr[A = 1| L_1, \ldots, L_K] = 1 / \{1 + \exp(-\gamma_0 - \gamma_1 L_1 - \ldots - \gamma_K L_K)\}$, where $\gamma_0, \ldots, \gamma_K$ are parameter values designed for each scenario. Intercept γ_0 was adjusted so that the specified exposure proportion (20% or 50%) was achieved on average.

Table S3. Parameter values for outcome models in the simulation.

Number of Confounders	Risk ratio $\exp(\beta_2)$ …, $\exp(\beta_{K+1})$																			
	L_1	L_2	L_3	L_4	L_5	L_6	L_7	L_8	L_9	L_{10}	L_{11}	L_{12}	L_{13}	L_{14}	L_{15}	L_{16}	L_{17}	L_{18}	L_{19}	L_{20}
5	0.5	2	1.2	0.9	2.5															
10	0.7	1.6	1.1	0.97	2.2	1.05	0.7	1.6	1.1	0.97										
20	0.8	1.2	1.02	0.99	1.3	1.01	0.8	1.2	1.02	0.99	1.3	1.01	0.8	1.2	1.02	0.99	1.3	1.01	0.8	1.2

The outcome models are log-binomial regression models: $\Pr[Y = 1 | A, L_1, \ldots, L_K] = \exp(\beta_0 + \beta_1 A + \beta_2 L_1 + \ldots + \beta_{K+1} L_K)$, where $\beta_0, \ldots, \beta_{K+1}$ are parameter values designed for each scenario. The risk ratio for exposure, $\exp(\beta_1)$, varied between 1, 1.3, and 2. Intercept β_0 was adjusted so that the specified outcome proportion (1%, 2%, 4%, 8%, or 16%) was achieved on average.

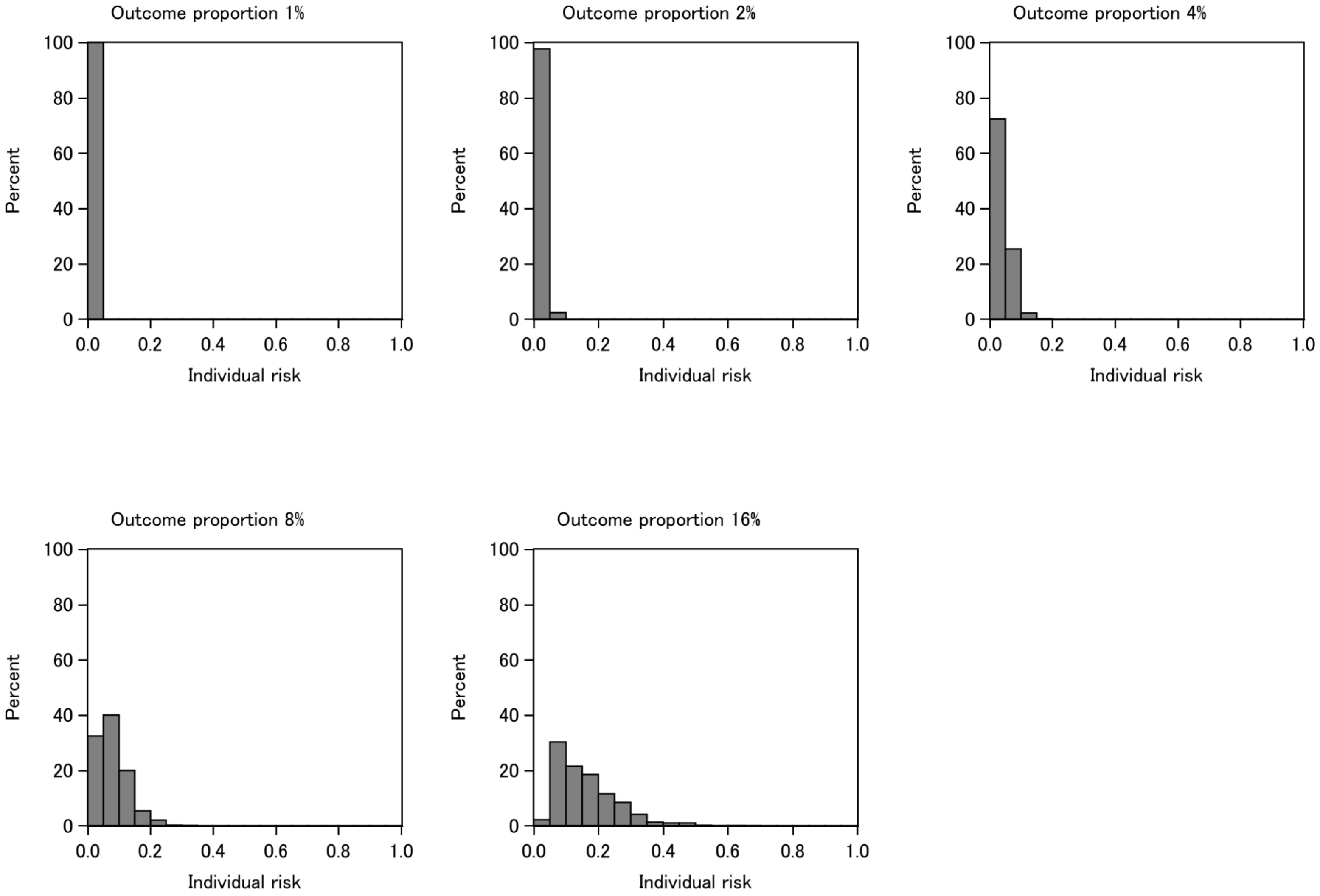

Fig. S1. Example distribution of individual risks for scenarios with 10 confounders, exposure proportion of 20%, and risk ratio of 1.3 .

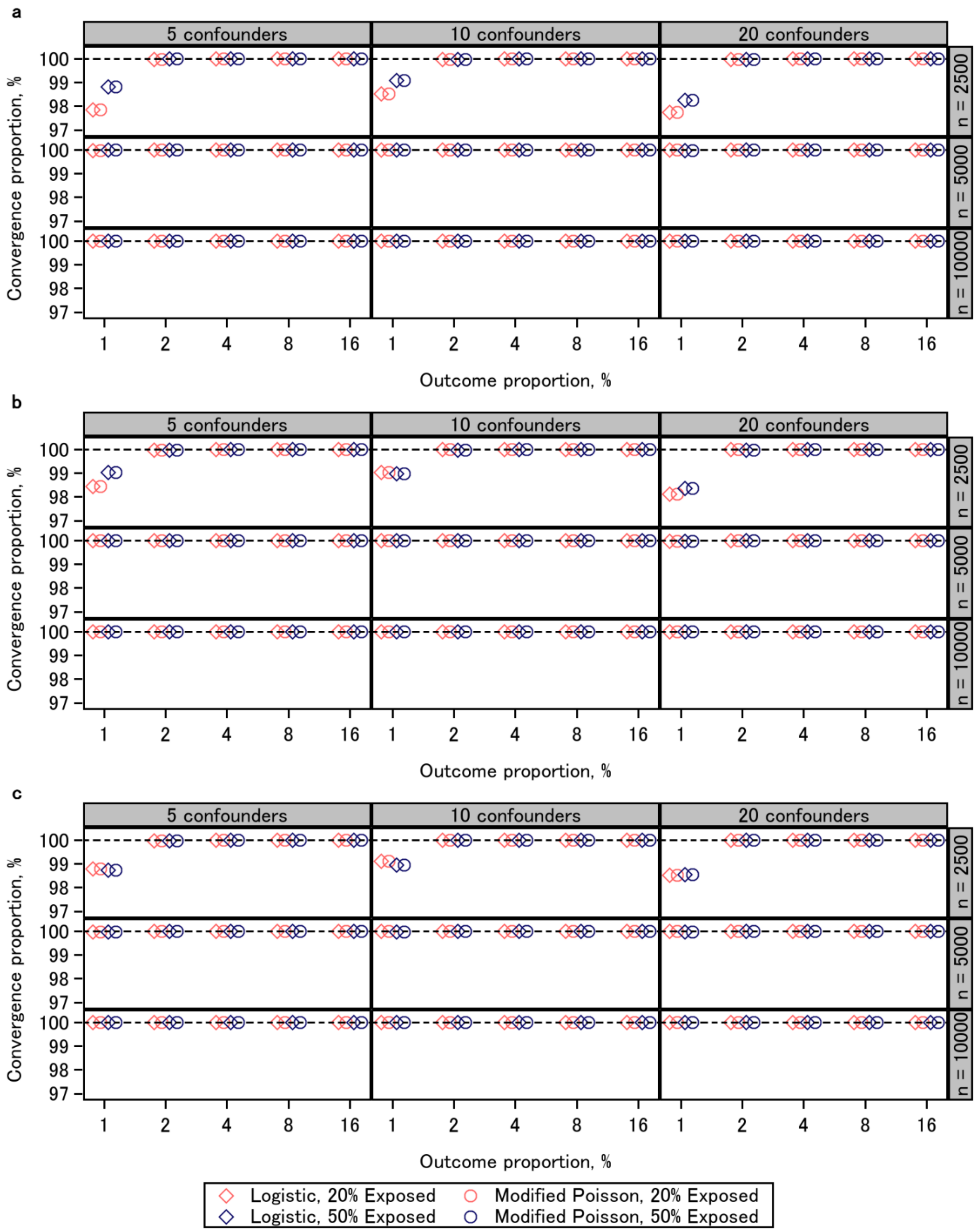

Fig. S2. Convergence proportion according to the number of confounders (columns) and the number of subjects (rows): **a** risk ratio 1; **b** risk ratio 1.3; **c** risk ratio 2.

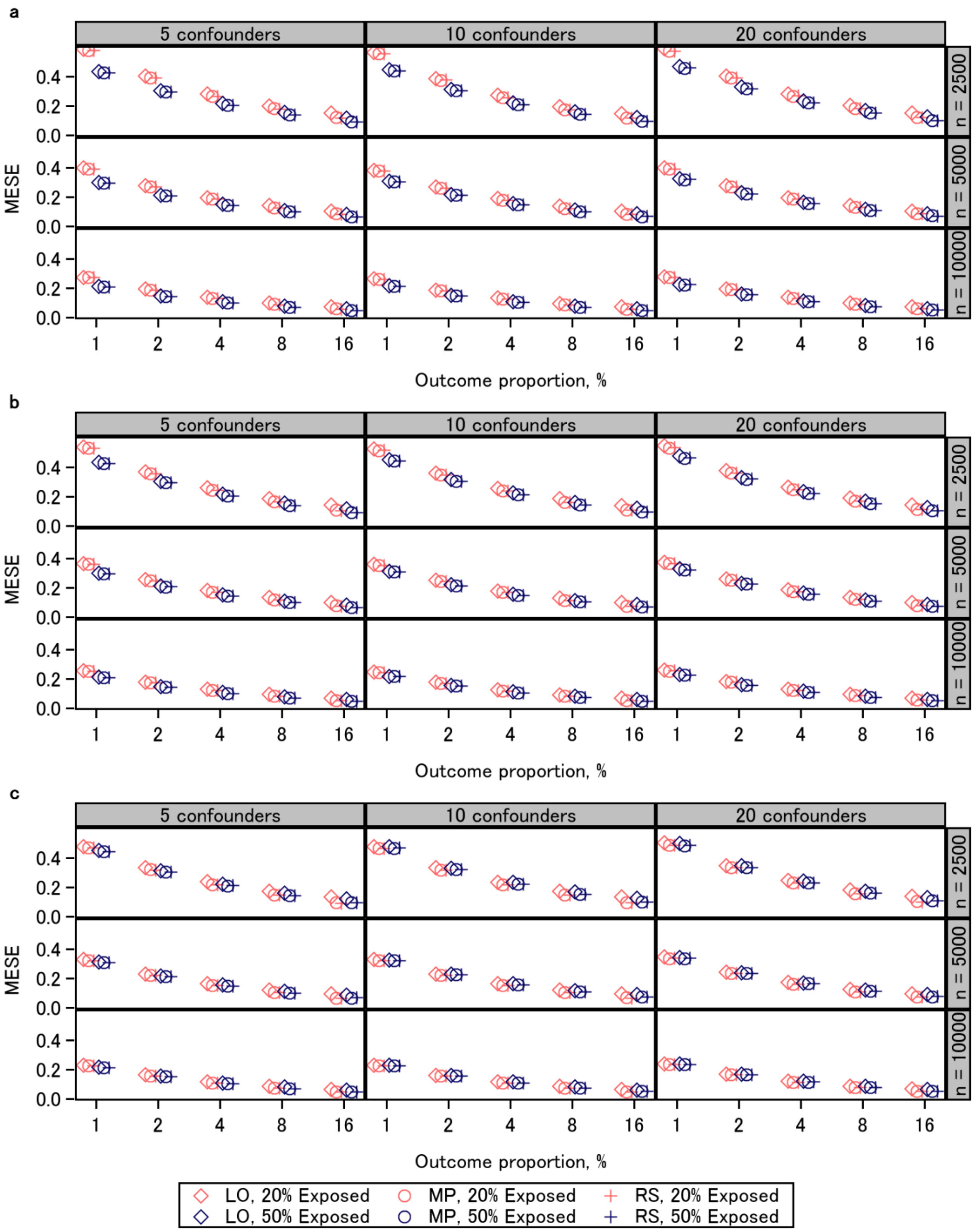

Fig. S3. Mean estimated standard error (MESE) according to the number of confounders (columns) and the number of subjects (rows): **a** risk ratio 1; **b** risk ratio 1.3; **c** risk ratio 2.

LO logistic regression; *MP* modified Poisson regression; *RS* regression standardization
