# Supplementary material for: A simulation study of regression approaches for estimating risk ratios in the presence of multiple confounders
Source: Emerg Themes Epidemiol. 2021 Dec 11;18:18. doi: 10.1186/s12982-021-00107-2 (PMC8665581; doi:10.1186/s12982-021-00107-2)
Supplement: Supplementary file 2 — Additional file 2. Additional Simulation Experiments. [file 12982_2021_107_MOESM2_ESM.pdf]

Additional file 2 to "A Simulation Study of Regression Approaches for Estimating Risk Ratios in the Presence of Multiple Confounders"

Description of the Additional Simulation Experiments

Table S4. Parameter values for outcome models in the additional simulation.

Number of Confounders	Risk ratio $\exp(\beta_2)$ …, $\exp(\beta_{K+1})$																			
	L_1	L_2	L_3	L_4	L_5	L_6	L_7	L_8	L_9	L_{10}	L_{11}	L_{12}	L_{13}	L_{14}	L_{15}	L_{16}	L_{17}	L_{18}	L_{19}	L_{20}
5	0.81	1.04	1.2	0.97	1.3															
10	0.81	1.04	1.2	0.97	1.3	0.93	0.81	1.04	1.2	0.97										
20	0.81	1.04	1.2	0.97	1.3	0.93	0.81	1.04	1.2	0.97	1.3	0.93	0.81	1.04	1.2	0.97	1.3	0.93	0.81	1.04

The outcome models are log-binomial regression models: $\Pr[Y = 1| A, L_1, \ldots, L_K] = \exp(\beta_0 + \beta_1 A + \beta_2 L_1 + \ldots + \beta_{K+1} L_K)$, where $\beta_0, \ldots, \beta_{K+1}$ are parameter values designed for each scenario. The risk ratio for exposure, $\exp(\beta_1)$, varied between 1, 1.3, and 2. Intercept β_0 was adjusted so that the specified outcome proportion (1%, 2%, 4%, 8%, or 16%) was achieved on average.

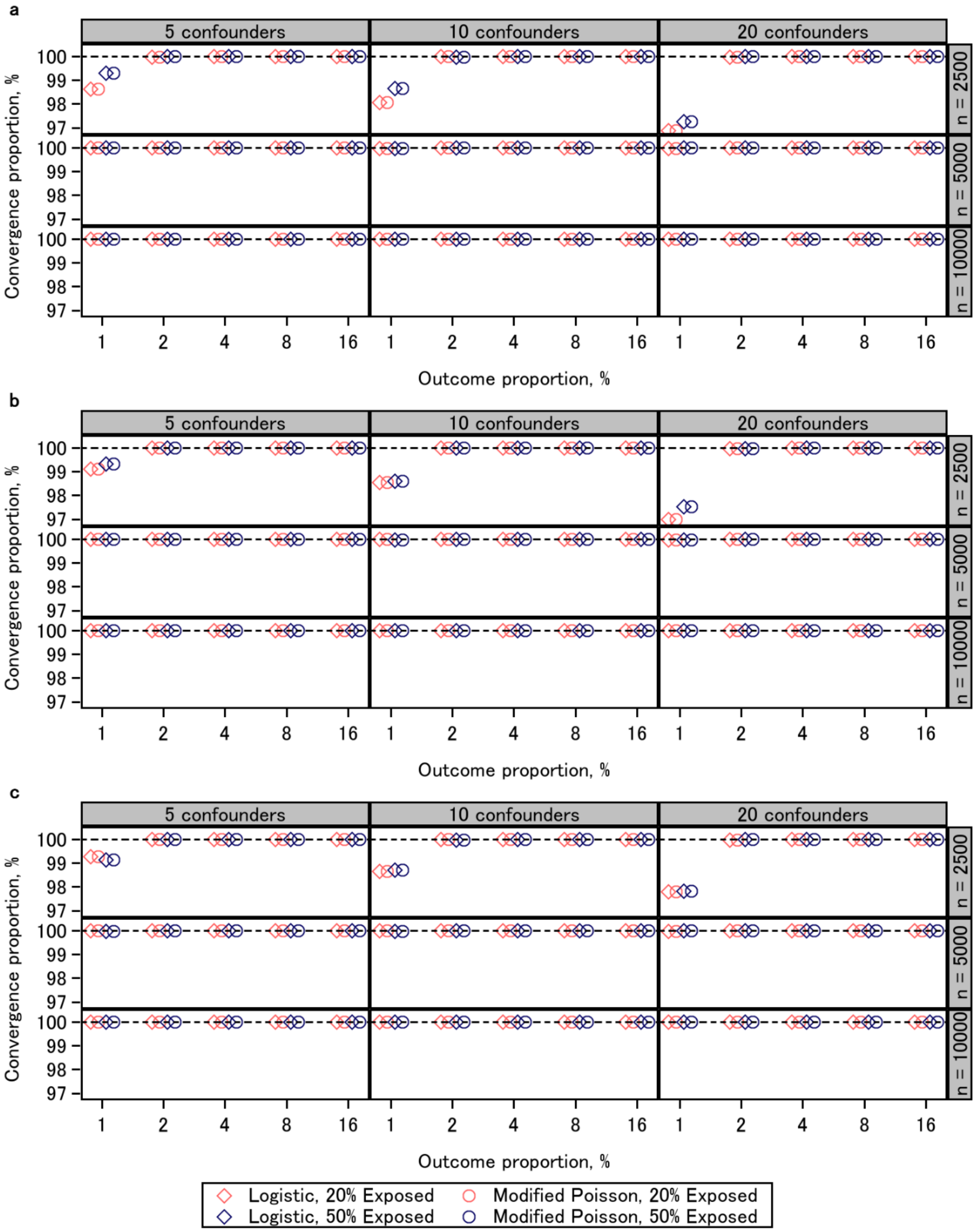

Fig. S4. Convergence proportion in the additional simulation according to the number of confounders (columns) and the number of subjects (rows): **a** risk ratio 1; **b** risk ratio 1.3; **c** risk ratio 2.

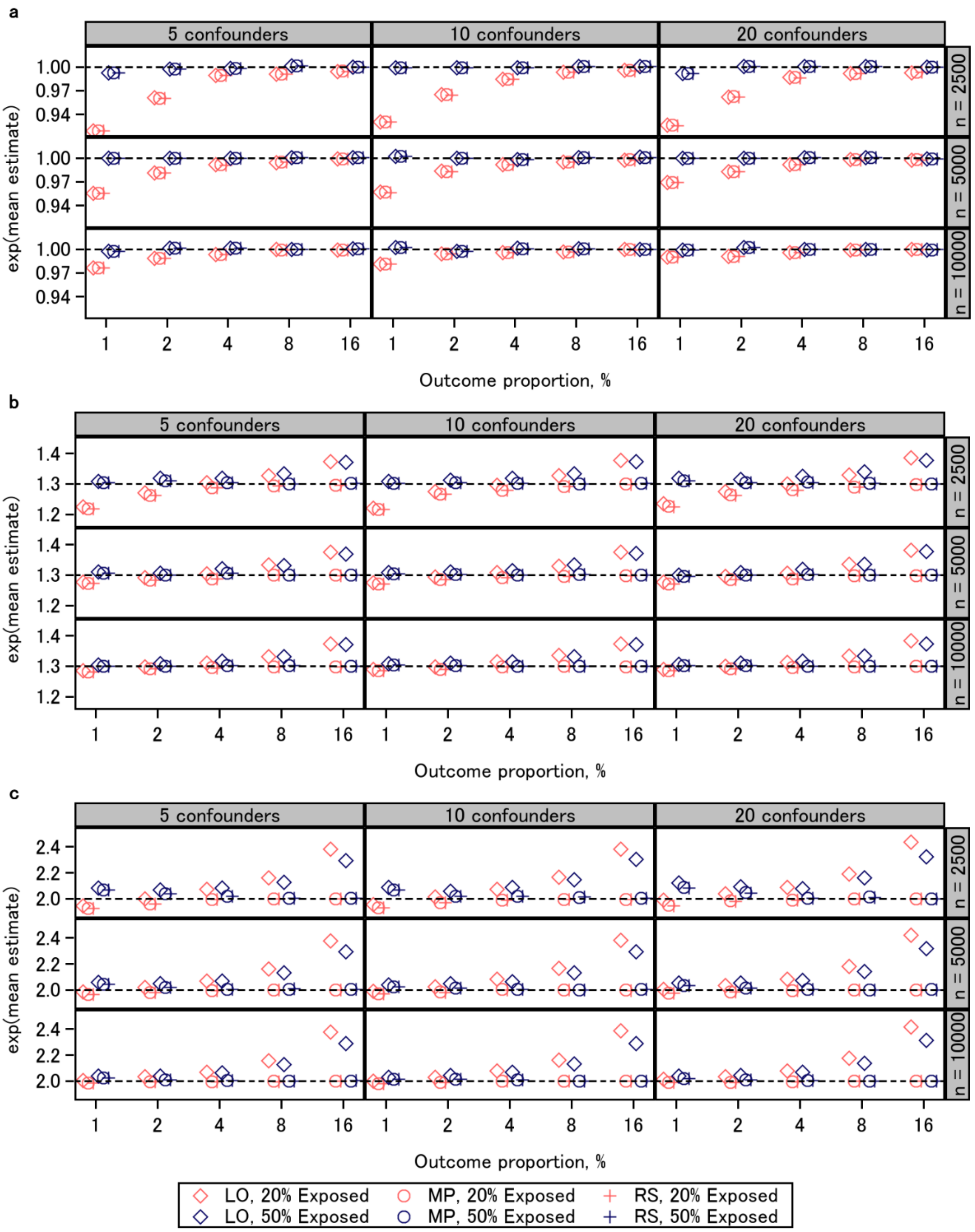

Fig. S5. Mean estimated log risk ratio transformed back to linear scale in the additional simulation according to the number of confounders (columns) and the number of subjects (rows): **a** risk ratio 1; **b** risk ratio 1.3; **c** risk ratio 2.

LO logistic regression; *MP* modified Poisson regression; *RS* regression standardization

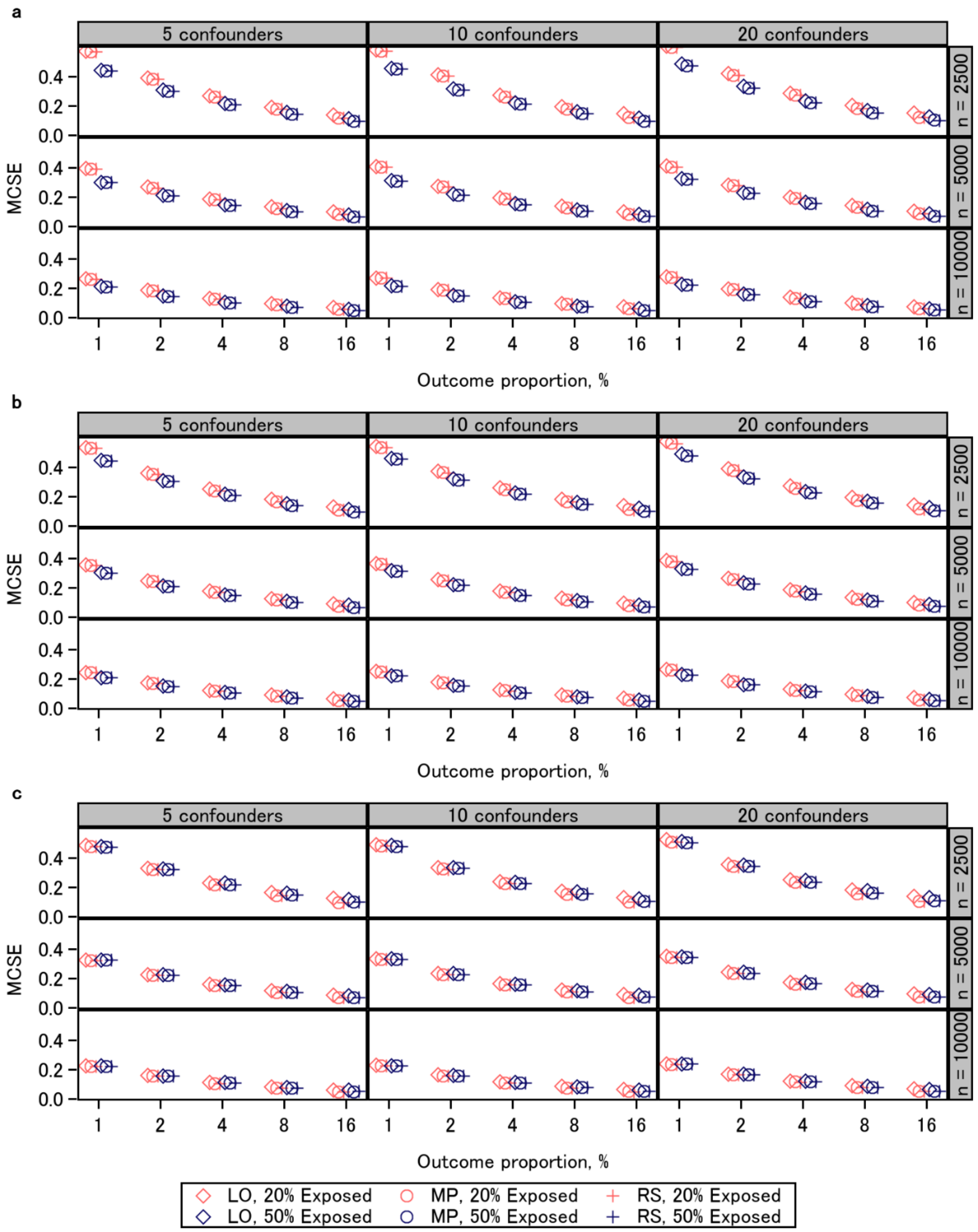

Fig. S6. Monte Carlo standard error (MCSE) in the additional simulation according to the number of confounders (columns) and the number of subjects (rows): **a** risk ratio 1; **b** risk ratio 1.3; **c** risk ratio 2.
LO logistic regression; *MP* modified Poisson regression; *RS* regression standardization

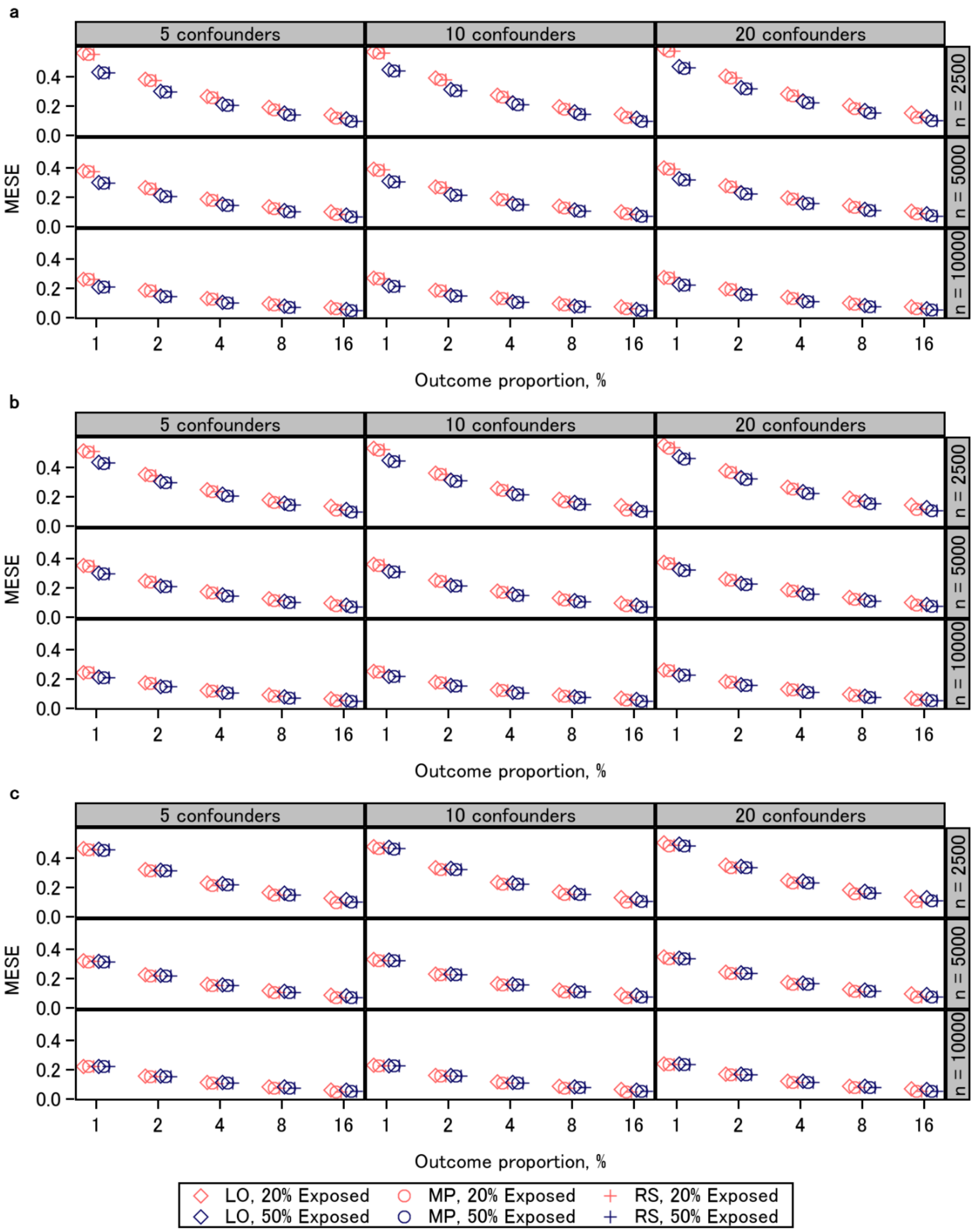

Fig. S7. Mean estimated standard error (MESE) in the additional simulation according to the number of confounders (columns) and the number of subjects (rows): **a** risk ratio 1; **b** risk ratio 1.3; **c** risk ratio 2.
LO logistic regression; *MP* modified Poisson regression; *RS* regression standardization

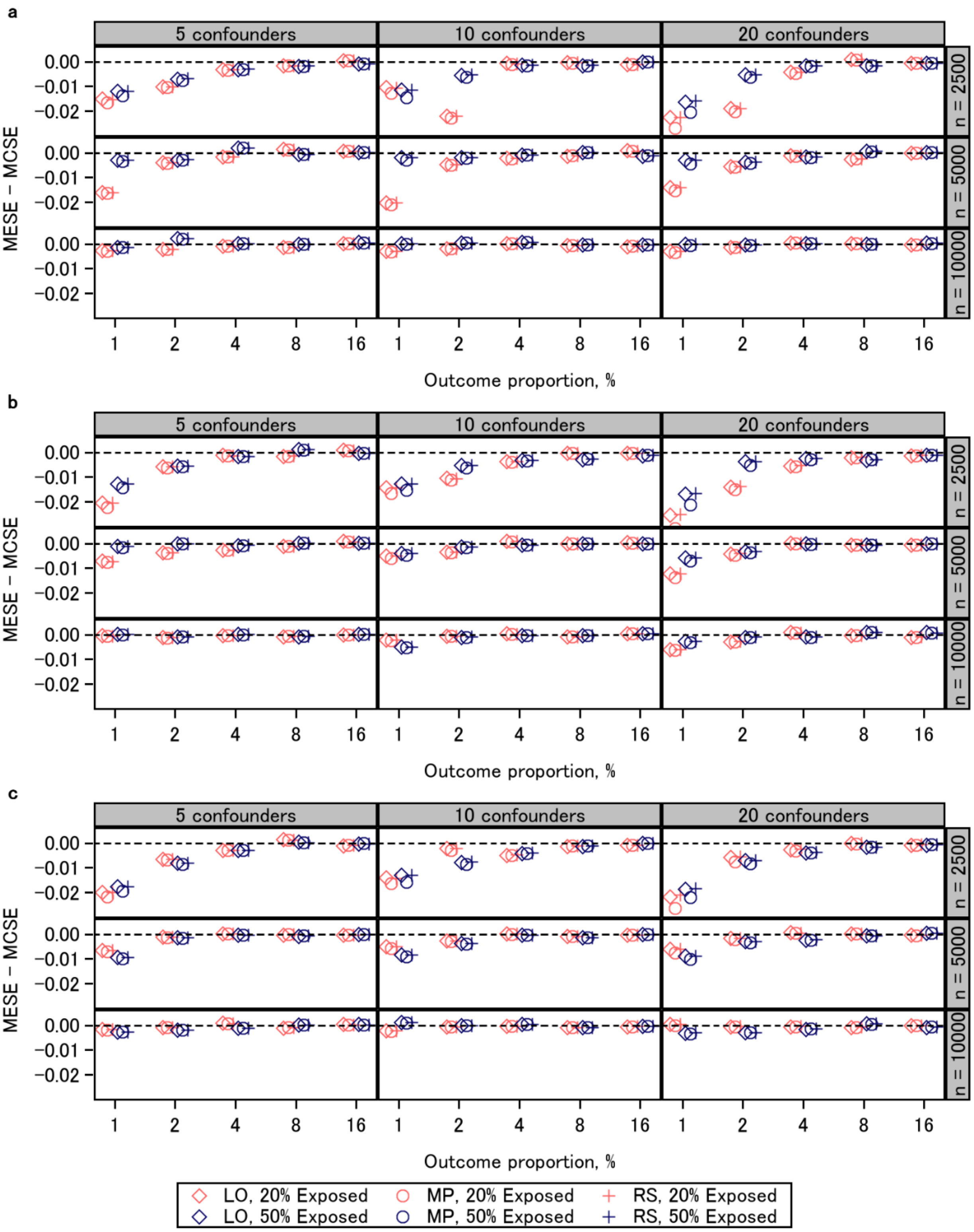

Fig. S8. Mean estimated standard error (MESE) minus Monte Carlo standard error (MCSE) in the additional simulation according to the number of confounders (columns) and the number of subjects (rows): **a** risk ratio 1; **b** risk ratio 1.3; **c** risk ratio 2.

LO logistic regression; *MP* modified Poisson regression; *RS* regression standardization

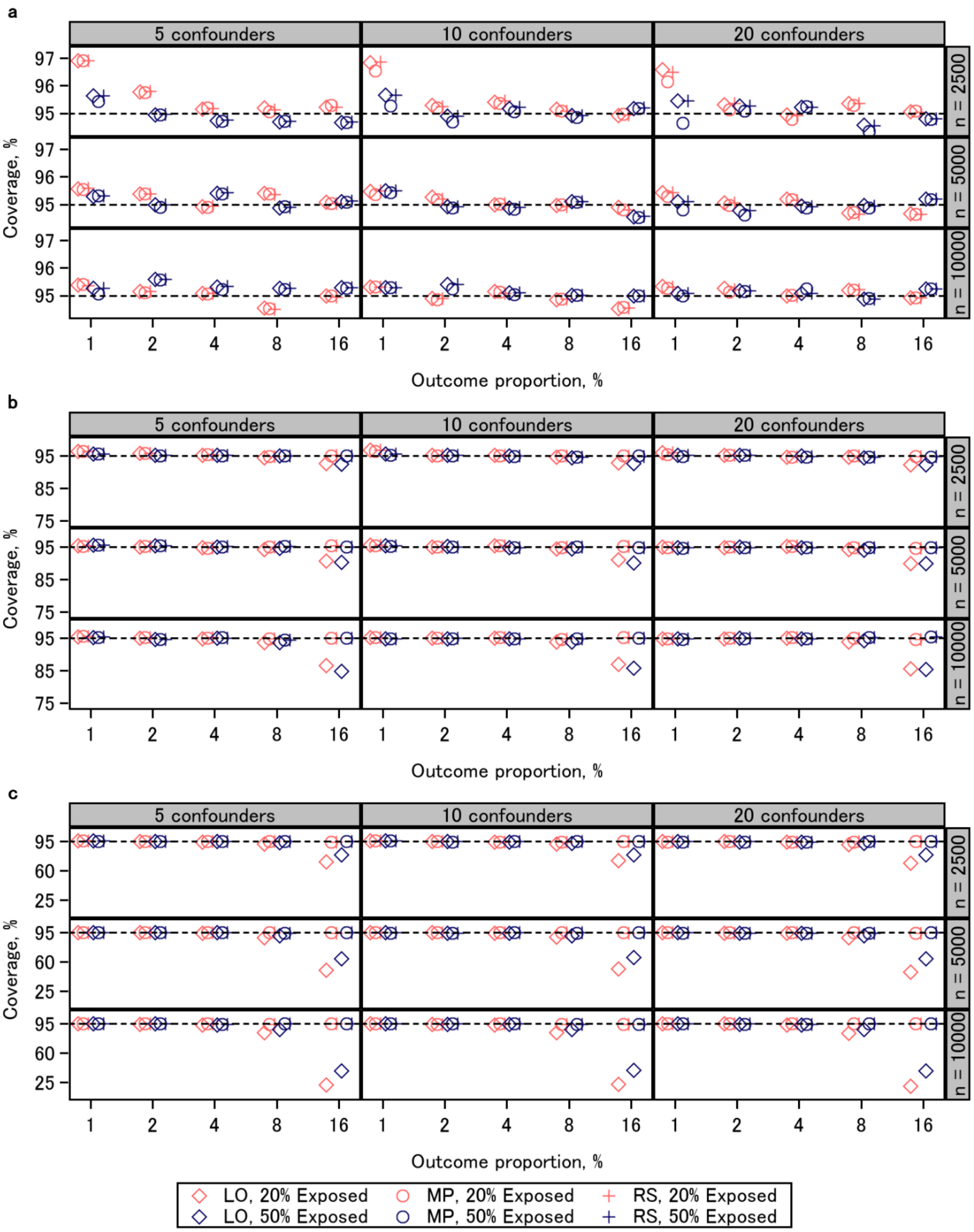

Fig. S9. Coverage probability of the 95% Wald confidence interval in the additional simulation according to the number of confounders (columns) and the number of subjects (rows): **a** risk ratio 1; **b** risk ratio 1.3; **c** risk ratio 2.

LO logistic regression; *MP* modified Poisson regression; *RS* regression standardization
